# The Pathology of Fatal Avian Malaria Due to *Plasmodium elongatum* (GRW6) and *Plasmodium matutinum* (LINN1) Infection in New Zealand Kiwi (*Apteryx* spp.)

**DOI:** 10.3390/ani12233376

**Published:** 2022-12-01

**Authors:** Emma Gulliver, Stuart Hunter, Laryssa Howe, Fernanda Castillo-Alcala

**Affiliations:** School of Veterinary Science, Massey University, Palmerston North 4410, New Zealand

**Keywords:** avian malaria, *Plasmodium*, kiwi, mortality, *elongatum*, GRW6, *matutinum*, LINN1

## Abstract

**Simple Summary:**

Avian malaria refers to the parasitic blood infection caused by species of *Plasmodium. Plasmodium* spp. have a worldwide distribution and records of mortality extend across a range of bird orders and species. In wild bird populations, infection is maintained within reservoir hosts, which are able to tolerate a chronic, low level of parasitism generally without severe health consequences. The introduction of these adapted avian species and their malarial parasites to naïve bird populations has resulted in devastating impacts on some non-adapted bird species, with the demise of Hawaiian amakihi as a pertinent example. In New Zealand, avian malaria was first recorded in introduced blackbirds (*Turdus merula*) in the 1920s, and reports of mortality in endemic and native bird species have been sporadic over the last three decades. Of the five species of kiwi, four are considered at risk or threatened by conservation standards. Here, we aim to describe the pathology of avian malaria as a cause of mortality in kiwi (*Apteryx* spp.) and the species of *Plasmodium* involved to further our understanding of this disease in arguably the most iconic of New Zealand’s endemic birds.

**Abstract:**

Avian malaria caused by *Plasmodium* species is a known cause of mortality in avifauna worldwide, however reports within New Zealand kiwi (*Apteryx* spp.) are scant. Postmortem reports from kiwi were obtained from the Massey University/Te Kunenga ki Pūrehuroa School of Veterinary Science Pathology Register from August 2010–August 2020. Gross lesions were described from postmortem reports, and archived H.E.-stained slides used for histological assessment. Nested PCR testing was performed on formalin-fixed paraffin-embedded tissue samples to assess the presence of *Plasmodium* spp. and *Toxoplasma gondii* DNA and cases with a PCR-positive result were sequenced to determine the lineage involved. Of 1005 postmortem reports, 23 cases of confirmed or suspected avian malaria were included in this study. The most consistent gross lesions included splenomegaly, hepatomegaly, and interstitial pneumonia with oedema. Histological lesions were characterised by severe interstitial pneumonia, pulmonary oedema, interstitial myocarditis, hepatic sinusoidal congestion and hypercellularity, and splenic macrophage hyperplasia and hyperaemia/congestion with numerous haemosiderophages. Cytoplasmic meronts were consistently found within endothelial cells of a variety of tissues, and within tissue macrophages of the liver, lung and spleen. A diagnosis of avian malaria was confirmed via PCR testing in 13 cases, with sequencing revealing *P. matutinum* (LINN1) and *P. elongatum* (GRW6) as the species involved. This is the largest case series describing the pathology of avian malaria as a cause of mortality in endemic New Zealand avifauna.

## 1. Introduction

Avian malaria is a disease caused by apicomplexan haemosporidian parasites of the genera *Plasmodium* and *Haemoproteus,* with other related genera of veterinary importance including *Leucocytozoon.* Collectively, these haemosporidian parasites are a significant cause of morbidity and mortality in a range of bird species worldwide. Avian malaria caused by *Plasmodium* spp. was first identified in blackbirds (*Turdus merula*) and song thrushes (*Turdus philomelos*) in New Zealand in the 1920s, with records of mortality events in several native and endemic bird species over the last few decades highlighting it as a potential emerging disease. In New Zealand, at least 17 *Plasmodium* spp. have been identified, infecting birds from a range of avian orders including native and introduced Psittacines, Passeriformes, Anseriformes and Coraciiformes [1,2,3,4,5,6,7,8,9,10]. Blackbirds are believed to be the main reservoir for infection, and at least two species of *Culex* mosquito vectors have been found in the North Island [11,12,13]. Only recently, mixed plasmodial infections were identified in several introduced avian species [14]. It should be noted that, to date, infection with other haemosporidian parasites in wild birds in New Zealand is limited to *Leucocytozoon* in marine penguins [15,16], and there are no reports of *Haemoproteus* in endemic avifauna.

*Plasmodium* spp. has an indirect lifecycle, with sexual development (sporogony) occurring within a mosquito vector followed by two asexual stages within the avian host, including merogony (exoerythrocytic and erythrocytic) and gametogony. The outcome of infection is a complex and incompletely understood balance between the degree of parasitism, species and lineage of *Plasmodium* and the immune response of the avian host [17]. In wild bird populations, infection is maintained within adapted (‘reservoir’), hosts, which are able to tolerate a chronic, low level of parasitism typically without severe health consequences. In contrast, infection of a non-adapted avian host may result in either failure of the lifecycle and successful clearance of organisms, or induction of severe immunopathology triggered by exoerythrocytic stages [18,19]; in the latter case, exoerythrocytic proliferation can be so overwhelming that fatality occurs before a significant erythrocytic parasitaemia is able to develop [20]. In live birds, avian malaria has traditionally been diagnosed by examination of a blood smear, in which erythrocytic merozoites and gametocytes can be visualized [21]. In fatal infections, diagnosis relies on demonstration of exoerythrocytic stages in the tissues alongside lesions most often characterised by splenomegaly, hepatomegaly and interstitial pneumonia. There is the potential for overlap of some of these lesions with other apicomplexan infections, particularly those caused by *Toxoplasma gondii* [22,23], however a combination of parasite morphology, location in the tissues and molecular diagnostics such as PCR, immunohistochemistry [24] or in situ hybridization [25] help to differentiate these. Determining the species and lineage of *Plasmodium* is now commonly done by with nested PCR and genetic sequencing [19,26], which differentiates between infection with other closely related Haemosporidia including *Haemoproteus* and *Leucocytozoon* genera.

Ongoing conservation efforts have led to improvements in the conservation status of the North Island brown kiwi (*A. mantelli*; NIBK) and little spotted kiwi (*A. owenii*), although the rowi (*A. rowii*), great spotted kiwi (*A. haastii*), and tokoeka (*A. australis*) continue to be threatened and have either stable or declining populations [27]. Mortality reports in kiwi have highlighted the major impact of predation in wild populations [28,29,30] but detailed studies on naturally occurring infectious disease are often limited to case reports. As a result, the relevance of avian malaria to the ongoing survival of kiwi is yet to be fully established and there are few reports in the literature. Organisms consistent with *Plasmodium* spp. were first reported in blood smears of two captive NIBK chicks at Auckland Zoo in 2000. One chick was treated with antimalarial medications, however both had organisms present in the blood three months later [31], although molecular testing to confirm this diagnosis was not reported. A study in 2012 described the death of a great spotted kiwi in Canterbury due to infection with *Plasmodium* spp. (LINN1) [6]. A second mortality event was reported in 2013 when a wild-born juvenile NIBK brought into captivity in Rotorua succumbed to systemic *Plasmodium (Huffia) elongatum* (GRW6) infection [7]. Subsequent surveillance testing identified *Plasmodium* spp. organisms in the blood smears of 25/32 juveniles at the same facility, though testing of numerous other individuals across an additional five facilities failed to find any further cases and no other deaths were reported [7]. Prevalence studies using PCR testing on blood samples from wild NIBK populations demonstrated 1/10 testing positive for *Plasmodium* spp. lineage GRW6 from Ponui Island [6] and 1/20 testing positive for *Plasmodium* spp. lineage LINN1 from Waimarino Forest [9], although no correlation with clinical disease was made.

The purpose of this study was to describe the lesions of avian malaria in kiwi and identify the species and lineage of *Plasmodium* involved to further our understanding of this disease within arguably the most iconic of New Zealand’s endemic birds.

## 2. Materials and Methods

Postmortem reports from all five species of kiwi were obtained from the Massey University/Te Kunenga ki Pūrehuroa School of Veterinary Science Pathology Register database from between August 2010 to August 2020. Reports in which a diagnosis of avian malaria due to *Plasmodium* spp. infection was made or suspected on the basis of gross and histopathological findings were included in the study. The species, age group, sex, captivity status and location were recorded for each kiwi, along with the year and month that the bird was submitted for postmortem examination. Age was classified as neonatal, juvenile, subadult or adult as recorded by the submitter. ‘Sanctuary’ was used to denote kiwi from outdoor locations with predator proof fencing. ‘Créche’ was used to denote juvenile kiwi being kept in a semi-managed outdoor facility.

Gross lesions were characterised based on the details included in the postmortem report, which was available for 22/23 cases. The body condition score from each bird was classified as ‘poor’, ‘suboptimal’ or ‘good’, based on the postmortem assessment of visceral and subcutaneous fat stores, pectoral muscle mass and bodyweight, as determined by the reporting pathologist. Histological assessment was carried out on archived Haematoxylin & Eosin (H&E)-stained slides, or slides prepared from archived formalin-fixed paraffin embedded (FFPE) tissues. Archival material and FFPE tissues were available for 21/23 cases, and histology reports were available for the remaining two cases. Selected tissue sections were examined with Perls Prussian blue iron stain or Martius Scarlett Blue fibrin stain to assess the presence of haemosiderin and fibrin thrombi, respectively.

FFPE tissues were selected for PCR analysis based on the presence of both organisms and lesions, where possible. Two kiwi with no gross or histologic evidence of apicomplexan infection were selected as additional negative controls. Six cases had previously tested PCR-positive for *Plasmodium* spp. on fresh (5) or formalin-fixed (1) tissues, and five of these had FFPE tissues available for repeat testing. One case of suspected avian malaria had no FFPE tissues available or previous test results to confirm the diagnosis.

DNA extraction was performed on 10 um scrolls of FFPE tissues using the manufacturer guidelines for a commercially available kit (Qiagen DNeasy^®^ Blood and Tissue kit, Valencia, CA, USA). Nested PCR was performed for *Plasmodium* spp. as described by Hellgren et al. [32], using primer sets HaemNF1/HaemNR3 and HaemF/HaemR to amplify a 480 bp fragment of DNA from the mitochondrial cytochrome b gene of avian malaria. Nested PCR for *Toxoplasma gondii* was performed on the same DNA extracts, using the method described by Aspinall et al. [33] and altered according to Roe et al. [34] with primer sets FOOD1/FOOD2 and FOOD3/FOOD4 to amplify a 400 bp fragment of DNA from the *Pppk-dhps* gene. The products from each nested PCR were electrophoresed on 1.5% agarose gel (UltraPure Agarose, Invitrogen, Carlsbad, CA, USA). Results were compared against known positive controls and negative controls. The DNA from each positive amplicon was purified using PureLink PCR purification kit (Invitrogen, Carlsbad, CA, USA) and sequenced using BigDye Terminator Version 3.1 Ready Analyser (Applied Biosystems Inc., Foster City, CA, USA). The sequence was examined for presence of overlapping peaks that may indicate co-infection and aligned using Geneious Primer (Biomatters, Auckland, New Zealand), and compared against published sequences in the National Centre of Biotechnology Information (NCDI) BLAST GenBank database. Representative isolates were submitted to the NCDI database.

Preliminary *T. gondii* genotyping was undertaken using the nested-PCR protocol described by Grigg & Boothroyd [35] to amplify the polymorphic B1 gene loci. The sequenced isolates were examined for single nucleotide polymorphisms at nucleotide 366 and 504 as described by Viscardi et al. [36] using Geneious to determine B1 genotype.

## 3. Results

Of 1005 postmortem reports between August 2010 and August 2020, a total of 23 cases of confirmed or suspected avian malaria were retrieved. The cause of death was determined to be avian malaria in 16 cases, concurrent avian malaria and toxoplasmosis in one case, possible avian malaria in three cases and multifactorial disease in three cases.

North Island brown kiwi comprised the majority of cases (16) with the remainder being little spotted kiwi (5) and great spotted kiwi (2). All age groups were represented, with one neonate, 14 juvenile, four subadult and four adult kiwi. Sex was listed as male in eight kiwi, female in 13 kiwi and unknown in two kiwi. Most cases originated from the North Island (21). Eight were from the wild, six were from sanctuaries, six were from créches and four were from captive sites. All cases were clustered over the late summer to early autumn period with five in the month of February, nine in March, eight in April and one in May (Table 1).

### 3.1. The Pathology of Avian Malaria in Kiwi

#### 3.1.1. Gross Lesions

**Figure 1:**
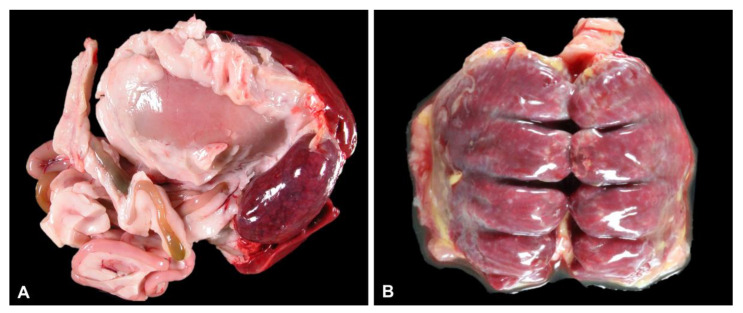
(**A**) Marked splenomegaly, with dark red to black parenchyma which was meaty on cut surface. (**B**) Dark red to purple, wet lungs which exuded fluid on cut surface.

The body condition score was noted as poor (6), suboptimal (7) or good (9) (Table S1). The most consistent gross finding was marked splenomegaly (17) characterised by rounding of splenic margins and a firm to meaty texture on cut surface (Figure 1A). One case had pinpoint to one mm diameter, coalescing tan foci scattered throughout the splenic parenchyma. In many cases, the lungs were dark red to purple (12), wet, heavy, and oozed clear red fluid or froth on cut surface (6) (Figure 1B). Hepatomegaly was mild to moderate (11) and the liver had an enhanced lobular pattern (5), pallor (5) or yellow-bronze discolouration (2). The liver of one bird had multifocal, small coalescing tan foci scattered throughout the parenchyma. Petechial haemorrhages were present over the epicardium of the cardiac apex or endocardium (5) or scattered throughout the lung (1). Ventral subcutaneous oedema was noted in three cases, all of which were in good body condition and only one of which had mild gastrointestinal parasitism. Adrenomegaly was noted in a single kiwi that was in poor body condition. Coelomic fluid was present in two kiwi, one of which had been frozen prior to postmortem examination (likely postmortem artefact).

Eleven kiwi were identified as having one or multiple comorbidities. Diagnoses included verminous ventriculitis (10/11), intestinal nematodiasis (4/11), intestinal coccidiosis (2/11), intestinal cestodiasis (3/11) and bacterial enteritis (2/11). Two of these also had lesions consistent with visceral (2) or neural (1) larval migrans. Eight of these kiwi were in suboptimal to poor body condition, four of which were noted to have heavy gastrointestinal parasitism. The three remaining kiwi were in good body condition and were all noted to have only mild gastrointestinal parasitism. The level of parasitism was not specified in the remaining cases.

#### 3.1.2. Microscopic Lesions

**Figure 2:**
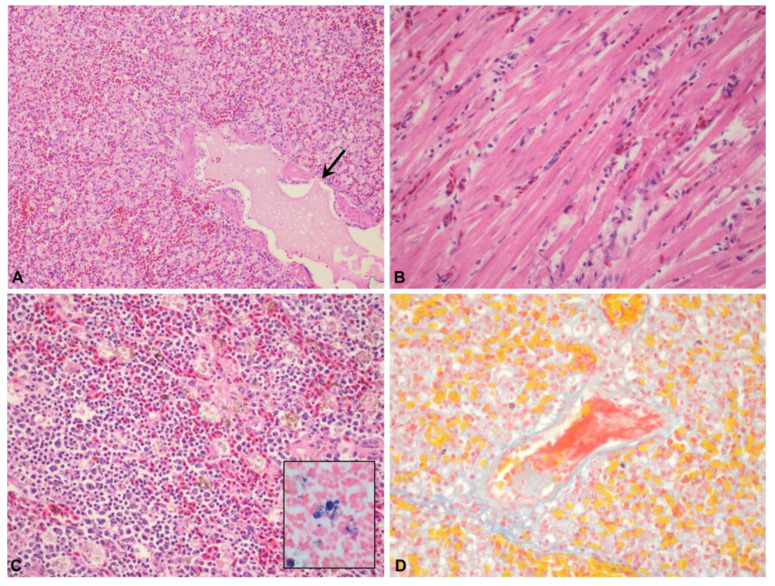
(**A**) Severe interstitial pneumonia with expansion of the interstitium with monocytic cells, granulocytes and proteinaceous fluid filling a parabronchus (arrow). H&E. (**B**) Expansion of the myocardial interstitium with macrophages and lesser granulocytes. H&E. (**C**) Expansion of the splenic parenchyma with increased numbers of tissue macrophages, frequently laden with brown cytoplasmic pigment. H&E. Pigment stained dark blue with Perls Prussian blue iron stain (inset), which is consistent with haemosiderin. (**D**) A fibrin thrombus (red) within the lumen of a medium calibre vessel in the lung, Martius Scarlett Blue stain.

Expansion of the parenchymal blood vasculature was the most prominent feature and was noted in the lung (21/21), spleen (20/23), liver (18/22), kidney (11/19), heart (9/21), adrenal gland (7/20) and brain (3/8). Areas of parenchymal congestion frequently had increased numbers of peripheralized monocytes within the vessel lumina. This was noted in the liver (15/22), lung (11/21), kidney (2/19), adrenal gland (1/10) and brain (1/8). Globular pale tan to brown cytoplasmic pigment within tissue macrophages was a common feature in congested organs and was seen in the spleen (15/23), lung (13/21) and liver (12/22). Perls Prussian Blue iron stain was done on the liver, lung and spleen of four cases showing that the pigment stained dark blue-black. The corresponding H&E sections were examined under polarised light and no birefringence of the pigment was seen. The pigment was interpreted as being consistent with haemosiderin (Figure 2C).

In the lung, interstitial inflammation was characterised by marked hypercellularity of the interstitium with moderate numbers of foamy macrophages and smaller numbers of lymphocytes and granulocytes (15/21), along with occasional localised deposits of smooth hypereosinophilic material (fibrin) and severe generalised congestion as previously described. Pulmonary oedema was characterised by pooling of proteinaceous fluid within air spaces and expansion of the interstitium with clear space (5/21) (Figure 2A). In the heart, interstitial myocarditis was characterised by separation of the cardiomyocytes with low to moderate numbers of macrophages/histiocytes and smaller numbers of granulocytes, typically concentrated around expanded blood vessels (13/21) (Figure 2B). Mild interstitial and epicardial haemorrhage were evident in some sections of heart (2/21), though not all in which haemorrhage was reported as a gross lesion, likely due to variation in trimming. The splenic parenchyma had increased numbers of tissue macrophages (hyperplasia) (11/23) and occasionally had areas of fibrin pooling and oedema (6/23). Within the liver, portal areas were moderately expanded with mixed lymphocytes, plasma cells and foamy macrophages, often with infiltration into adjacent sinusoids (16/22). Aggregates of amorphous to fibrillar, hypereosinophilic material that stained bright red with Martius Scarlett Blue were present in small to medium calibre vessels in the lung (4/21), kidney (3/19), adrenal gland (1/10) and brain (3/8) indicating the presence of fibrin thrombi (Figure 2D). Rarely, small foci of coagulative necrosis were scattered within the splenic parenchyma (3/23) and pulmonary interstitium (2/21), and one kiwi had mild centrilobular hepatic necrosis. 

**Figure 3:**
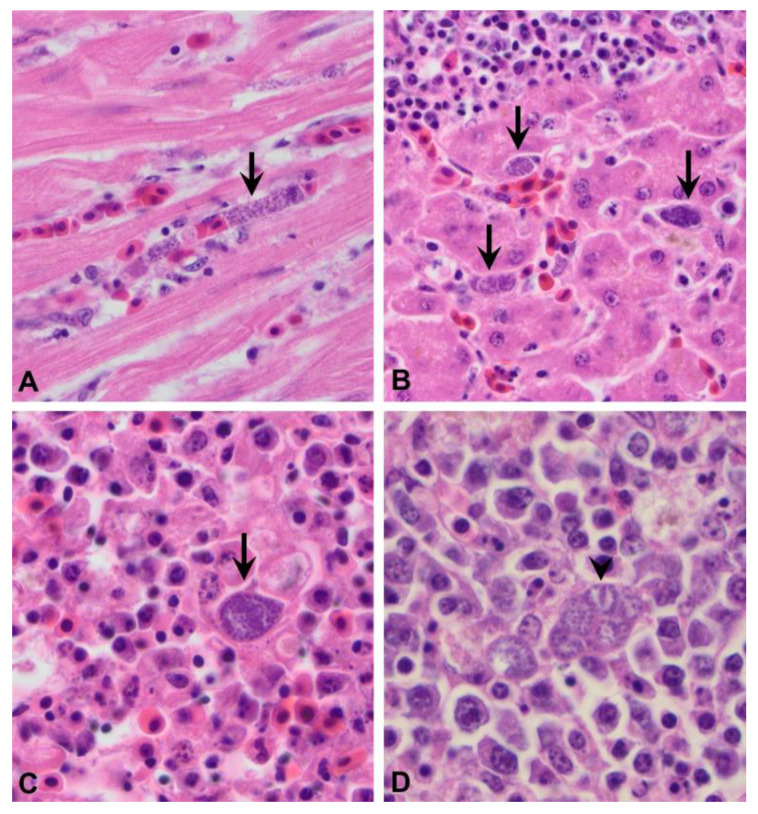
Exoerythrocytic meronts (arrow) of *Plasmodium matutinum* (LINN1) within capillary endothelial cells in the heart (**A**), Kupffer cells or endothelial cells of the liver (**B**) and a macrophage in the spleen (**C**); multiple exoerythrocytic meronts (arrowhead) of *Plasmodium elongatum* (GRW6) within a macrophage in the spleen (**D**). H&E.

In H&E tissue sections, organisms with morphological features consistent with *Plasmodium* species were positively identified in the spleen (22/23), lung (20/21), liver (20/22), heart (17/21), kidney (14/19), gastrointestinal tract (6/13) and adrenal gland (6/10). Organisms were not identified in any of the examined brain sections (0/8). Organisms were consistently found within capillary endothelial cells and within Kupffer cells of the liver and macrophages within the spleen (Figure 3A–C). With light microscopy, organisms were characterised by a single cytoplasmic meront measuring approximately 20–30 um diameter and filled with numerous <1 um diameter, round, stippled, basophilic merozoites. Splenic macrophages occasionally contained up to three or four cytoplasmic meronts (Figure 3D). Rarely, organisms were found within hepatocytes and in these cases would occasionally consist of two or three meronts.

A summary of gross and histologic lesions along with distribution of organisms in the tissues is presented in Table S1. 

### 3.2. Molecular Findings

Of the 21 tested cases, nine were PCR-positive for *Plasmodium* spp. using FFPE tissues, including two that had previously tested PCR-positive. One of these kiwi tested PCR-negative on the first assay and PCR-positive on a second assay, using freshly prepared samples of the same FFPE tissues for each test. Genetic sequencing of the positive amplicons revealed 100% similarity with known sequences from GenBank, and included *Plasmodium matutinum* (LINN1)(GenBank GQ471953; *n* = 5) and *Plasmodium elongatum* (GRW6)(GenBank DQ368381; *n* = 4), including a previously submitted *P. elongatum* (GRW6) isolate from a North Island brown kiwi (GenBank HQ454000). Representative sequences from the isolates of *P. matutinum* (LINN1) from a great spotted kiwi and a little spotted kiwi were submitted to GenBank (GenBank OP783971 and OP783970, respectively). 

Of the 12 tested cases which were PCR-negative for *Plasmodium* in this assay, three had previously tested PCR-positive using fresh tissues, including the single kiwi that had concurrently tested PCR-positive for *T. gondii*. This kiwi tested weakly PCR-positive for *T. gondii* with the current assay. Four kiwi tested PCR-positive for *T. gondii* only and sequencing confirmed the presence of *T. gondii pppk-dhps* gene DNA (100% sequence homology, GenBank U81497). Preliminary genotyping of the *B1* gene revealed a Type I genotype at these loci. Five cases were PCR-negative for both agents. 

Considering current and previous PCR-assay results, a diagnosis of avian malaria was confirmed in 13 cases, all of which had an original diagnosis of avian malaria based on gross and histological lesions. A summary of the tested tissues, sequencing and PCR results is presented in Table 2.

## 4. Discussion

Most cases of avian malaria in kiwi were seen in the North Island of New Zealand, with a geographic distribution consistent with areas known to be inhabited by the affected species of kiwi [37] along with mosquito vectors [11,12]. There was a seasonal spread in the warmer months from February to May, which along with the spatial distribution correlates with warmer regions of temperate New Zealand and when vector numbers are maximal. Many reports on non-adapted hosts, particularly penguins, have shown a strong correlation between disease and warmer months over spring and summer [38,39,40,41,42,43], although morbidity may also occur during autumn and winter months, particularly in migratory birds [44].

Superficially, the isolated geography of New Zealand would suggest that kiwi are more likely to lie on the non-adapted end of the spectrum of susceptibility to *Plasmodium* spp., but there is still much about infection in wild avifauna that is not known. Owing to the protected status of kiwi, prospective infection studies are not possible. Human contact with non-captive kiwi is not allowed during breeding season between June to December, which significantly impedes epidemiological surveillance in some populations. There may also be interspecies differences in kiwi susceptibility, as NIBK are more numerous and probably more genetically diverse than smaller kiwi populations, including little spotted kiwi. This could partially explain previous reports of parasitaemia in wild-born captive juvenile NIBK [7,31] or PCR-positivity of blood from wild adult NIBK [6,9] without a link with mortality, but limited data on the immunological response prevents further interpretation of the significance of these results. Captive kiwi are faced with a unique set of challenges, as placement in outdoor enclosures may increase exposure to vectors and reservoir hosts, and may be further impacted on by various stressors. It is therefore difficult to cast general assumptions about how well adapted the different kiwi populations are to infection with *Plasmodium* spp. Nevertheless, the cases presented here stand to highlight the potential for a fatal outcome, and it may at least be considered a sporadic cause of death in kiwi. In captive populations, netting of enclosures to reduce contact with mosquito vectors is warranted. 

As part of ongoing conservation efforts, many neonatal kiwi are raised in captivity before being released back into the wild once they reach a bodyweight at which they are considered less likely to be predated [45]. Increased physiological stress and movement between geographic areas has been implicated in avian malaria outbreaks in captive birds, including kiwi [7], and is particularly well described in penguins where disease is often associated with movement of birds into captive rehabilitation facilities [39]. As kiwi tend to breed between June and December, higher numbers of immuno-naïve juvenile kiwi exist at times when insect vectors are plentiful over summer. It is noteworthy that, in this study, avian malaria also resulted in the death of adult and subadult kiwi, one of which had only mild concurrent gastrointestinal parasitism as a comorbidity, highlighting that even apparently healthy and mature kiwi may succumb to infection. Breeding status may play a role in immunomodulation and increased susceptibility to infection through altered host metabolism, and has been suggested as a factor in malaria of Hawaiian amakihi (*Chlorodrepanis virens*) where infected females and males have been demonstrated to have higher serum prolactin and testosterone levels, respectively, compared to uninfected birds [46]. Nine of the 13 kiwi that tested PCR-positive for malaria were female, which included two of the adult birds. This is in contrast to some reports which highlight increased susceptibility of male birds including tawny pipits (*Anthus campestris*), nutmeg mannikins (*Lonchura punctulata*) and Iiwi (*Vestiaria coccinea*) [47,48], although this may reflect differing biology between avian species. The influence of breeding season on susceptibility of adult kiwi is uncertain, as the breeding status of adult birds at time of death is unknown from these reports. 

Poor body condition has been associated with increased susceptibility to avian malarial infection and reduced survival time in some birds [47,48], and a greater degree of parasitaemia has been associated with higher serum corticosterone concentrations in chronically infected lowland amakihi [46]. Kiwi that were in poor body condition for a variety of reasons, including severe gastrointestinal parasitism, may have been affected by a degree of immunosuppression and hence were more susceptible to infection. One kiwi in poor body condition had adrenomegaly which likely reflects hypertrophy due to chronic physiological stress. However, the majority of kiwi did not have significant comorbidities and many that died were in good body condition. These kiwi invariably had abundant malarial organisms throughout the tissues, reflecting the preponderance for extensive spread of exoerythrocytic stages that tends to occur in non-adapted hosts. 

Cell-mediated immunity plays a major role in elimination of organisms following acute infection and is primarily facilitated by monocyte-macrophage lineage cells in the haematopoietic organs. In adapted hosts, chronic infection may occur with development of acquired immunity and is associated with a decline in parasitaemia, antibody production and persistence of organisms in monocyte-macrophage lineage cells, often without significant ill effect [49,50,51]. Development of tolerance or resilience to chronic infection has been suggested to involve host diversity in genes encoding major histocompatibility complex molecules, which are involved in antigen-presentation in both cell-mediated and humoral immune responses [52]. This may have particular relevance to smaller bird populations where genetic diversity is lower [52] and hence non-adapted birds are more likely to succumb to the acute stages of infection, although this is an incompletely understood realm in many avian species, including kiwi. 

The most consistent lesions were severe interstitial pneumonia with pulmonary oedema, splenomegaly with macrophage hyperplasia and expansion of the red pulp by congestion or hyperaemia, hepatomegaly with mononuclear infiltration of portal areas and sinusoids, as well as increased numbers of circulating monocytes within the blood vasculature, which was noted in a variety of tissues. There is little information pertaining to the precise pathogenesis of interstitial pneumonia in avian malaria infections, although it is a common finding in reports of mortality in experimentally infected Hawaiian amakihi [53], blackbirds [6] and several species of penguin including Magellanic penguins (*Spheniscus magellanicus*) [39], yellow-eyed penguins (*Megadyptes antipodes*) [10], little penguins (*Eudyptula minor*) [40], a Fiordland crested penguin (*Eudyptes pachyrhynchus*) [8] and black-footed penguins (*Spheniscus demersus*) [54]. Some reports describe a more granulocytic pulmonary infiltrate than what was seen here [39,53]. In *P. falciparum* infection in humans, haematogenous spread of merozoites and the associated systemic inflammatory response lead to alterations in cell surface adhesion molecules on infected erythrocytes and capillary endothelial cells, resulting in increased adhesion, increased vascular permeability and leakage of plasma proteins into the alveolar spaces and interstitium [55]; while mammalian and avian malaria differ in many respects, a similar process may be happening in these kiwi. Enlargement of the spleen and liver are also well documented [38,39,40,41,42,47,53,54,56] and reflect altered haemodynamics, erythrocyte sequestration and proliferation of monocyte-macrophage lineage cells.

The cause of death was likely respiratory failure and hypoxaemia secondary to severe pulmonary pathology induced by the exoerythrocytic proliferation of *Plasmodium* spp. For some kiwi, interstitial myocarditis may have contributed to impaired cardiorespiratory function, although the degree of cardiac inflammation was relatively mild when compared to the lung. Severe anaemia and circulatory shock have been cited as a cause of death in acute infections, which typically peaks around one week post-infection with the onset of parasitaemia [43] and is primarily due to extravascular haemolysis mediated by the spleen, a process which is particularly well described in human malaria [57]. While there was evidence of extravascular erythrophagy in the spleen, liver and lung, the absence of antemortem haematological data made the presence and severity of anaemia difficult to assess in these kiwi. Obstruction of cerebral capillaries with exoerythrocytic meronts has also been suggested as a cause of death in some passerine hosts [3,19]. In this study, meronts were found in endothelial cells and macrophages within all the available tissues examined except for the brain, although cerebral intravascular fibrin thrombi were seen in cerebral capillaries in three cases. The presence of intravascular fibrin thrombi presumably reflects a combination of activation of the coagulation cascade secondary to endothelial damage and systemic immune stimulation and may have contributed to morbidity. Foci of necrosis were small to locally extensive and seen within the spleen or liver of five birds, four of which were PCR-positive for *Plasmodium* spp. These may have resulted from organism-induced tissue necrosis, hypoxia or potentially ischaemia secondary to fibrin thrombosis, although the cause was not clear from histological examination.

*Plasmodium* spp. lineage LINN1 was first reported in New Zealand in 2012 [5,6] and subsequently characterised as *Plasmodium* (subgenus *Haemamoeba*) *matutinum* (LINN1) in 2017 [58]. *Plasmodium* (subgenus *Huffia*) *elongatum* (GRW6) is a generalist parasite with low host specificity and following characterization in 2008 [59], has been reported in several surveys on wild avifauna throughout New Zealand [4,5,6,9,14]. Both *Plasmodium* lineages have a widespread worldwide distribution and have been implicated as a cause of pathology across a range of avian hosts [19,38,39,41,60,61]. Lesions and organism morphology did not differ markedly between cases produced by either agent. 

One kiwi (#5) had no tissues or slides available for examination, hence the diagnosis of malaria could not be further investigated. Three cases (#3, #4 and #8) that had previously tested PCR-positive for *Plasmodium* spp. using fresh tissues tested negative in this study with FFPE tissues. There were five kiwi that tested negative in both *Plasmodium* spp. and *T. gondii* PCR assays. Four of these kiwi (#9, #13, #14 and #16) had lesions and intracellular meronts consistent with *Plasmodium* spp. and are considered to be false negative results. The fifth (#7) had lesions suggestive of possible malaria but no organisms were seen in tissue section, hence it is probably unlikely that this kiwi died of avian malaria, although the possibility of prior resolving disease cannot be excluded. Formalin fixation compromises DNA integrity through formation of cross linkages, which is worsened with longer periods of fixation [62]. Long periods of storage once embedded in paraffin may allow further opportunity for degradation [62,63] and lead to false negative results. While use of nested PCR typically increases test sensitivity, some genotypes may be missed depending on methodology including primer selection. It has also been suggested that the presence of large amounts of avian host DNA in highly congested tissues may have a dilutional effect on parasite DNA [64], but this is difficult to assess quantitatively with light microscopy. 

There were four kiwi which tested PCR-positive for only *T. gondii.* One kiwi was suspected to have succumbed to multifactorial disease including starvation and gastrointestinal parasitism (#11), and another had lesions suggestive of apicomplexan infection but was autolysed (#1), which may have impacted on assessment of some histological features. The significance of avian malaria infection to the death of these two birds is therefore uncertain. In the other two kiwi (#12 and #21), the lesions and morphology of organisms were indistinguishable from the confirmed cases of avian malaria. These kiwi did not have foci of parenchymal necrosis, the main lesion associated with toxoplasmosis, and organisms were visible in both endothelial cells and tissue macrophages, which is not a usual feature of natural *T. gondii* infections [22,24,65]. *T. gondii* tissue cysts and zoites may be slightly larger than *Plasmodium* spp. [22], and tend to show tropism for muscle and the central nervous system, with tissue cysts generally most readily visible within cardiomyocytes and the brain of diseased birds [23,24]. Neural lesions were not present in the three of these kiwi that had brain available for examination and organisms were seen only within endothelial cells and not cardiomyocytes in the heart. Studies on the prevalence of *T. gondii* exposure in kiwi are limited [66], and a study on New Zealand raptors using similar PCR methodology on FFPE tissues demonstrated positive *T. gondii* and *Plasmodium* spp. DNA amplification in the absence of visible organisms or lesions within the tissues [67]. In both studies, correlation of *T. gondii* positivity with compatible lesions was not made. The presentation of these kiwi (#12 and #21) remains suggestive of avian malaria; immunohistochemistry would be required to further ascertain the significance of these results but was unfortunately outside the scope of this study. The morphology and location of organisms was not consistent with other apicomplexan parasites including *Atoxoplasma, (Isospora), Haemoproteus* and *Leucocytozoon* hence these were not considered for molecular testing. In the authors’ experience, *Haemoproteus* has not been found in endemic avifauna samples submitted to our laboratory since surveillance for haemoparasites began in 2006.

## 5. Conclusions

This is the largest case series linking molecular data with the lesions of avian malaria in an endemic bird of New Zealand and expands our knowledge of infection with *Plasmodium matutinum* (LINN1) and *Plasmodium elongatum* (GRW6) as a cause of mortality in kiwi. The most consistent lesions include splenomegaly with macrophage hyperplasia, severe interstitial pneumonia with pulmonary oedema and interstitial myocarditis. Exoerythrocytic meronts were widespread and found within the cytoplasm of endothelial cells throughout the body, in tissue macrophages of the spleen and lung, and in Kupffer cells of the liver. Mortality is likely attributable to cardiorespiratory failure; whether kiwi which succumbed to infection survived long enough to develop anaemia as a comorbidity was uncertain. Several factors impair studies of natural disease in kiwi, however further investigation surrounding the host response could potentially be gleaned from monitoring captive birds in the North Island during peak transmission times.

## Figures and Tables

**Table 1:** Summary of case signalment, temporal data, captivity status, location and cause of death in kiwi (*Apteryx* spp.) submitted to Massey University/Te Kunenga ki Pūrehuroa School of Veterinary Science Pathology service between August 2010 and August 2020, with a diagnosis of confirmed or suspected avian malaria.

Case (#)	Species	Age	Sex	Year	Month	Captivity Status	Location	Recorded Cause of Death
1	NIBK	Neonate	M	2010	March	Wild	Northland	PM
2 *	GSK	Juvenile	M	2010	April	Captive	Canterbury	AM
3 **	NIBK	Juvenile	F	2011	April	Captive	Bay of Plenty	AM
4	GSK	Juvenile	F	2012	April	Créche	West Coast	AM
5	NIBK	Juvenile	F	2012	March	Wild	Auckland	PM
6	NIBK	Adult	UK	2013	February	Captive	Waikato	AM
7	NIBK	Subadult	M	2014	February	Wild	Northland	PM
8	NIBK	Juvenile	F	2014	March	Créche	Hawke’s Bay	AM Toxoplasmosis
9	NIBK	Subadult	M	2014	March	Wild	Waikato	AM
10	NIBK	Juvenile	M	2015	April	Créche	Hawke’s Bay	AM
11	NIBK	Juvenile	F	2016	March	Sanctuary	Hawke’s Bay	MF
12	NIBK	Juvenile	M	2017	February	Créche	Hawke’s Bay	AM
13	NIBK	Juvenile	UK	2017	February	Créche	Hawke’s Bay	MF
14	NIBK	Juvenile	F	2017	March	Créche	Hawke’s Bay	MF
15	NIBK	Juvenile	F	2018	February	Wild	Waikato	AM
16	LSK	Adult	F	2018	April	Sanctuary	Auckland	AM
17	LSK	Subadult	F	2018	April	Sanctuary	Auckland	AM
18	LSK	Subadult	M	2018	April	Sanctuary	Auckland	AM
19	LSK	Adult	F	2018	April	Sanctuary	Auckland	AM
20	LSK	Adult	F	2018	May	Sanctuary	Auckland	AM
21	NIBK	Juvenile	M	2019	March	Wild	Hawke’s Bay	AM
22	NIBK	Juvenile	F	2020	March	Captive	Hawke’s Bay	AM
23	NIBK	Juvenile	F	2020	March	Wild	Bay of Plenty	AM

* This case was described in reference [6]. ** This case was described in reference [7]. NIBK, North Island brown kiwi (*Apteryx mantelli*); GSK, Great spotted kiwi (*A. haastii*); LSK, Little spotted kiwi (*A. owenii*); F, female; M, male; UK, unknown sex; AM, avian malaria; MF, multifactorial cause of death including suspected avian malaria; PM, lesions suggestive of possible avian malaria.

**Table 2:** Results of *Plasmodium* spp. PCR, *Plasmodium* spp. sequencing and *Toxoplasma gondii* PCR for all cases.

Case (#)	Previous Test Results	Tissues Tested	*Plasmodium* spp. PCR	*Plasmodium* spp. Sequencing	*Toxoplasma gondii* PCR
1	N/A	Liver, heart	Negative		Positive
2	Positive–*Plasmodium* spp. (LINN1) on fresh liver *	Heart, spleen, lung, thymus	Positive	*P. matutinum* (LINN1) ^1^	Negative
3	Positive–*Plasmodium* spp. on fresh spleen **	Liver, spleen, lung, adrenal gland	Negative		Negative
4	Positive–*Plasmodium* spp. (AFTUR5/LINN1) on fresh liver	Spleen	Negative		Negative
5	N/A	None available	N/D		N/D
6	Positive–*P. elongatum* on spleen	None available	N/D		N/D
7	N/A	Liver, lung, spleen	Negative		Negative
8	Positive–*P. elongatum* and *T. gondii* on fresh lung and spleen	Liver, lung	Negative		Weak Positive
9	N/A	Liver	Negative		Negative
10	Positive–*P. elongatum* on formalin-fixed heart	Liver, heart	Positive	*P. elongatum* (GRW6) ^2^	Negative
11	N/A	Spleen, lung, bursa	Negative		Positive
12	N/A	Heart, spleen, liver	Negative		Positive
13	N/A	Lung, liver, heart, spleen	Negative		Negative
14	N/A	Lung, spleen, liver, kidney	Negative		Negative
15	N/A	Spleen, liver, lung, adrenal gland	Positive	*P. elongatum* (GRW6) ^2^	Negative
16	N/A	Liver, spleen, lung	Negative		Negative
17	N/A	Lung, heart	Positive	*P. matutinum* (LINN1) ^3^	Negative
18	N/A	Lung, spleen, liver	Positive	*P. matutinum* (LINN1) ^3^	Negative
19	N/A	Spleen, liver, kidney, heart	Positive	*P. matutinum* (LINN1) ^3^	Negative
20	N/A	Lung, liver, spleen, kidney	Positive	*P. matutinum* (LINN1) ^3^	Negative
21	N/A	Spleen, liver, lung	Negative		Positive
22	N/A	Spleen, liver, kidney, lung	Positive	*P. elongatum* (GRW6) ^2^	Negative
23	N/A	Liver, spleen, lung	Positive	*P. elongatum* (GRW6) ^2^	Negative

* This was described in reference [6]. ** This was described in reference [7]. N/A, not applicable. N/D, not done. ^1^ GenBank OP783971. ^2^ Genbank HQ454000. ^3^ Genbank OP783970.

## Data Availability

Not applicable.

## Supplementary Materials

The following supporting information can be downloaded at: https://www.mdpi.com/article/10.3390/ani12233376/s1, Table S1: Summary of gross lesions, tissues available for histological assessment, histological lesions and distribution of apicomplexan organisms in kiwi (*Apteryx* spp.) with a confirmed or suspected diagnosis of avian malaria.
