# Supplementary material for: The Pathology of Fatal Avian Malaria Due to Plasmodium elongatum (GRW6) and Plasmodium matutinum (LINN1) Infection in New Zealand Kiwi (Apteryx spp.)
Source: Animals (Basel). 2022 Dec 1;12(23):3376. doi: 10.3390/ani12233376 (PMC9740581; doi:10.3390/ani12233376)
Supplement: Supplementary file 1 [file animals-12-03376-s001.zip › animals-2044044-supplementary.pdf]

animals

MDPI

Supplementary Material

The pathology of fatal avian malaria due to *Plasmodium elongatum* (GRW6) and *Plasmodium matutinum* (LINN1) infection in New Zealand kiwi (Apteryx spp.)

Emma Gulliver[1*], Stuart Hunter[2], Laryssa Howe[3] and Fernanda Castillo-Alcala[4]

[1] School of Veterinary Science, Massey University, New Zealand
[2] School of Veterinary Science, Massey University, New Zealand
[3] School of Veterinary Science, Massey University, New Zealand
[4] School of Veterinary Science, Massey University, New Zealand
* Correspondence: emma.gulliver@outlook.com

Simple Summary: Avian malaria refers to the parasitic blood infection caused by species of *Plasmodium. Plasmodium* species have a worldwide distribution and records of mortality extend across a range of bird orders and species. In wild bird populations, infection is maintained within reservoir hosts, which are able to tolerate a chronic, low level of parasitism generally without severe health consequences. The introduction of these adapted avian species and their malarial parasites to naïve bird populations has resulted in devastating impacts on some non-adapted bird species, with the demise of lowland Hawaiian amakihi as a pertinent example. In New Zealand, avian malaria was first recorded in introduced blackbirds (*Turdus merula*) in the 1920s, and reports of mortality in endemic and native bird species have been sporadic over the last three decades. Of the five species of kiwi, four are considered at risk or threatened by conservation standards. Here, we aim to describe the pathology of avian malaria as a cause of mortality in kiwi (*Apteryx* spp.) and the species of *Plasmodium* involved to further our understanding of this disease in arguably the most iconic of New Zealand's endemic birds.

Abstract: Avian malaria, caused by *Plasmodium* species, is a known cause of mortality in avifauna worldwide, however reports within New Zealand kiwi (*Apteryx* spp.) are scant. Postmortem reports from kiwi were obtained from the Massey University/Te Kunenga ki Pūrehuroa School of Veterinary Science Pathology Register from August 2010-August 2020. Gross lesions were described from postmortem reports, and archived H.E.-stained slides used for histological assessment. Nested PCR testing was performed on formalin-fixed paraffin-embedded tissue samples to assess the presence of *Plasmodium* species and *Toxoplasma gondii* DNA and cases with a PCR-positive *Plasmodium* species result were sequenced to determine the lineage involved. Of 1005 postmortem reports, 23 cases of confirmed or suspected avian malaria were included in this study. The most consistent gross lesions included splenomegaly, hepatomegaly, and interstitial pneumonia with oedema. Histological lesions were characterised by severe interstitial pneumonia, pulmonary oedema, myocarditis, hepatic sinusoidal congestion and hypercellularity, and splenic histiocytosis and hyperaemia/congestion with numerous haemosiderophages. Cytoplasmic meronts were consistently found within endothelial cells of a variety of tissues, and within tissue macrophages of the liver, lung and spleen. A diagnosis of avian malaria was confirmed via PCR testing in 13 cases, with sequencing revealing *P. matutinum* (LINN1) and *P. elongatum* (GRW6) as the species involved. This is the first case series describing the pathology of avian malaria as a cause of mortality in New Zealand avifauna.

Keywords: avian malaria; *Plasmodium*; kiwi; mortality; *elongatum;* GRW6; *matutinum;* LINN1

animals

MDPI

Supplementary Table S1. Summary of gross lesions, tissues available for histological assessment, histological lesions and distribution of apicomplexan organisms in kiwi (*Apteryx* spp.) with a confirmed or suspected diagnosis of avian malaria.

Case	Body condition	Gross lesions	Tissues available for histological assessment	Histological lesions	Distribution of organisms
1	Good	Free coelomic fluid	Heart, lung, liver, kidney, spleen, proventriculus, ventriculus, intestine, adrenal gland, brain.	Autolysed. Congestion in the lung, liver and spleen.	Abundant in spleen and lung. Occasional in heart, liver, kidney, gut and adrenal gland.
2*	Suboptimal	Spleen large and friable. Liver enlarged and friable. Lungs red and wet. Epicardial and endocardial haemorrhages.	Heart, lung, liver, kidney, spleen, ventriculus, intestine, brain, adrenal gland.	Interstitial myocarditis and epicardial haemorrhage. Interstitial pneumonia with congestion and foci of necrosis. Splenic congestion and fibrin pooling. Portal hepatitis, sinusoidal congestion and hypercellularity with scattered pigment-laden Kupffer cells. Congestion in the kidney and adrenal gland.	Abundant in heart, lung, liver and spleen. Occasional in kidney, gut and adrenal gland.
3**	Good	Spleen enlarged, dark and meaty. Liver enlarged and pale. Epicardial haemorrhages.	Heart, lung, liver, kidney, spleen, ventriculus, intestine, brain, adrenal gland.	Interstitial pneumonia with congestion, vascular hypercellularity and scattered pigment-laden macrophages. Splenic congestion and macrophage hyperplasia with scattered pigment-laden macrophages. Portal hepatitis and sinusoidal congestion and hypercellularity. Hypercellularity in myocardial vessels. Fibrin thrombi in small vessels of the lung, sinusoids of the liver and capillaries of the heart.	Abundant in lung, liver and spleen. Occasional in heart, kidney, gut and adrenal gland.
4	Good	Spleen enlarged and meaty. Pale kidneys.	Heart, lung, liver, spleen, ventriculus, intestine, brain.	Interstitial myocarditis. Congestion in the lung and brain. Splenic congestion with scattered pigment-laden macrophages and fibrin pooling. Portal hepatitis, sinusoidal congestion and hypercellularity. Rare fibrin thrombi in capillaries in the lung.	Abundant in the heart and spleen. Moderate in the lung and liver. Occasional in the gut.
5	Suboptimal	Spleen enlarged. Liver enlarged and pale.	None available.	Report only. Interstitial pneumonia. Splenomegaly with macrophage hyperplasia. Hepatomegaly with congestion, sinusoidal hypercellularity and scattered centrilobular necrosis.	Report only. Organisms not seen.

6	Unknown	No report available.	None available.	Report only. Splenomegaly with marked congestion and intracytoplasmic pigment.	Report only. Abundant in spleen.
7	Good	Spleen enlarged. Tan foci scattered throughout liver.	Heart, lung, liver, kidney, spleen, proventriculus, intestine.	Autolysed. Interstitial myocarditis with congestion. Pulmonary congestion and possible interstitial pneumonia. Portal hepatitis and sinusoidal congestion. Congestion of the spleen and kidney.	Organisms not seen
8	Poor	Spleen enlarged, meaty and red/black. Liver enlarged and bronze. Lungs dark purple.	Heart, lung, liver, kidney, spleen, proventriculus, ventriculus, intestine, brain, adrenal gland.	Autolysed. Interstitial myocarditis. Interstitial pneumonia with congestion, oedema and vascular hypercellularity. Splenic congestion and macrophage hyperplasia with scattered pigment-laden macrophages. Portal hepatitis and sinusoidal congestion. Hypercellularity in renal vasculature. Occasional fibrin thrombi in glomerular capillaries, interstitial renal vessels, and brain capillaries.	Moderate in the spleen. Occasional in the heart, lung, liver and adrenal gland.
9	Good	Spleen enlarged and meaty with scattered tan foci. Liver enlarged and pale. Lungs dark red.	Heart, lung, liver, kidney, spleen, proventriculus, ventriculus, intestine, brain, adrenal gland.	Autolysed. Interstitial myocarditis. Interstitial pneumonia with congestion. Splenic congestion with fibrin pooling and foci of necrosis. Portal hepatitis and sinusoidal congestion and hypercellularity. Congestion of the adrenal gland. Fibrin thrombi in small vessels and capillaries in the brain.	Moderate in the liver, spleen and lung. Occasional in the heart and kidney.
10	Poor	Lungs dark red and wet. Adrenomegaly.	Heart, lung, liver, kidney, spleen, proventriculus, ventriculus, intestine.	Interstitial myocarditis with congestion. Interstitial pneumonia with congestion and hypercellularity of vasculature. Splenic congestion and macrophage hyperplasia with scattered pigment-laden macrophages. Portal hepatitis, sinusoidal congestion and hypercellularity, scattered pigment-laden Kupffer cells and foci of necrosis.	Abundant in the heart. Moderate in the lung, liver and spleen. Occasional in the kidney and gut.
11	Poor	Free coelomic fluid.	Heart, lung, liver, kidney, spleen, ventriculus, intestine, brain, adrenal gland.	Interstitial pneumonia with congestion and oedema. Marked splenic congestion with frequent pigment-laden macrophages. Portal hepatitis with hypercellularity of sinusoids and scattered pigment-laden macrophages.	Occasional in the heart, lung and spleen.

12	Suboptimal	Spleen enlarged and meaty.	Heart, lung, liver, kidney, spleen, ventriculus, brain.	Autolysed. Interstitial myocarditis with congestion. Interstitial pneumonia with congestion and hypercellularity of vessels, oedema and pigment-laden macrophages. Splenic congestion, macrophage hyperplasia and fibrin pooling with abundant pigment-laden macrophages. Hepatic and renal congestion.	Occasional in the lung, liver, kidney and spleen.
13	Suboptimal	Spleen enlarged.	Heart, lung, liver, spleen.	Autolysed. Myocardial congestion and vascular hypercellularity. Congestion and scattered pigment-laden macrophages in the lung and spleen.	Moderate in the heart and liver. Occasional in the lung and spleen.
14	Poor	No report available.	Heart, lung, liver, kidney, spleen, proventriculus, ventriculus.	Interstitial pneumonia with congestion and pigment-laden macrophages. Splenic congestion with pigment-laden macrophages. Hepatic sinusoidal congestion and hypercellularity.	Abundant in the liver and spleen. Moderate in the heart, lung and kidney.
15	Suboptimal	Spleen enlarged and red/purple. Liver enlarged and red/yellow. Epicardial haemorrhages.	Heart, lung, liver, kidney, spleen, brain.	Pulmonary congestion and vascular hypercellularity with scattered haemosiderophages. Portal hepatitis and sinusoidal congestion and hypercellularity with scattered haemosiderophages. Splenic macrophage hyperplasia with scattered haemosiderophages. Congestion and fibrin thrombi in low calibre vessels and capillaries of the brain. Renal congestion.	Abundant in the lung, liver and spleen. Occasional in the heart and kidney.
16	Poor	Spleen enlarged and firm. Liver pale with lobular pattern. Lungs dark red.	Heart, lung, liver, kidney, spleen, brain, adrenal gland.	Autolysed. Interstitial pneumonia with congestion, vascular hypercellularity and scattered pigment-laden macrophages. Congestion of the spleen, heart, kidney and adrenal gland. Pigment within renal tubular epithelial cells.	Abundant in the heart. Moderate in the liver. Occasional in the spleen, lung, kidney and adrenal gland.
17	Good	Spleen enlarged and meaty. Liver enlarged and pale with granular capsule. Lungs dark red and wet. Subcutaneous oedema.	Heart, lung, liver, kidney, spleen, brain, adrenal gland.	Autolysed. Interstitial pneumonia with congestion, vascular hypercellularity and scattered pigment-laden macrophages. Interstitial myocarditis with vascular hypercellularity. Portal hepatitis with scattered pigment-laden Kupffer cells. Splenic congestion and macrophage hyperplasia with pigment-laden macrophages, fibrin pooling and foci of necrosis. Congestion of the adrenal gland.	Abundant in the heart. Occasional in the lung and spleen.

18	Poor	Spleen enlarged. Liver enlarged with lobular pattern. Lungs dark red/purple.	Heart, lung, liver, liver, kidney, spleen.	Interstitial pneumonia with congestion and scattered haemosiderophages. Interstitial myocarditis with congestion and vascular hypercellularity. Splenic congestion and macrophage hyperplasia with fibrin pooling and scattered haemosiderophages. Portal hepatitis, sinusoidal congestion and hypercellularity with scattered haemosiderophages. Rare fibrin thrombi in capillaries of the lung.	Abundant in the heart, liver and spleen. Moderate in the lung and kidney.
19	Good	Subcutaneous oedema.	Heart, lung, liver, kidney, spleen.	Autolysed. Interstitial pneumonia with congestion and scattered pigment-laden macrophages. Interstitial myocarditis with congestion and vascular hypercellularity. Splenic congestion and macrophage hyperplasia with scattered pigment-laden macrophages. Portal hepatitis, sinusoidal congestion and hypercellularity with scattered pigment-laden Kupffer cells.	Abundant in the heart. Moderate in the spleen and kidney. Occasional in the lung and liver.
20	Good	Spleen enlarged. Liver enlarged with lobular pattern. Lungs red and wet. Epicardial haemorrhages. Subcutaneous oedema.	Heart, lung, liver, kidney, spleen, ventriculus.	Interstitial pneumonia with congestion and scattered haemosiderophages. Interstitial myocarditis with congestion and vascular hypercellularity. Splenic congestion and macrophage hyperplasia with scattered haemosiderophages, fibrin pooling and foci of necrosis. Portal hepatitis, sinusoidal congestion and hypercellularity with scattered haemosiderophages.	Abundant in the heart. Occasional in the lung, kidney, spleen and gut.
21	Suboptimal	Spleen enlarged and meaty. Liver enlarged, red and mottled. Lungs dark red. Epicardial haemorrhages.	Heart, lung, liver, kidney, spleen, adrenal gland.	Pulmonary congestion with vascular hypercellularity and scattered pigment-laden macrophages. Myocardial congestion with vascular hypercellularity and interstitial haemorrhage. Congestion of the spleen and adrenal gland. Portal hepatitis, sinusoidal congestion and hypercellularity with scattered pigment-laden Kupffer cells.	Abundant in the lung, liver and spleen.

22	Suboptimal	Spleen enlarged. Lobular pattern in liver. Lungs red and wet.	Heart, lung, liver, kidney, spleen, adrenal gland.	Autolysed. Interstitial pneumonia with congestion and oedema, vascular hypercellularity and scattered pigment-laden macrophages. Interstitial myocarditis with vascular hypercellularity. Splenic congestion and macrophage hyperplasia with scattered pigment-laden macrophages. Portal hepatitis, sinusoidal congestion and hypercellularity with scattered pigment-laden Kupffer cells. Congestion of the kidney and adrenal gland. Plasmacytic interstitial nephritis. Fibrin thrombi in interstitial vessels in the adrenal gland.	Abundant in the liver. Moderate in the spleen. Occasional in the lung and adrenal gland.
23	Good	Spleen enlarged. Liver enlarged with lobular pattern. Lungs red and wet.	Heart, lung, liver, kidney, spleen, proventriculus, ventriculus, intestine.	Interstitial pneumonia with congestion and scattered haemosiderophages. Interstitial myocarditis. Splenic congestion and macrophage hyperplasia with scattered haemosiderophages. Hepatic sinusoidal congestion and hypercellularity with scattered haemosiderophages. Hypercellularity in renal vessels. Fibrin thrombus in a medium calibre vessel in the lung.	Abundant in the liver. Moderate in the spleen and heart. Occasional in the lung and kidney.

*This case was also described in [6]. **This case was also described in [7]. 1
